# Polypharmacy in older patients with chronic diseases: a cross-sectional analysis of factors associated with excessive polypharmacy

**DOI:** 10.1186/s12875-018-0795-5

**Published:** 2018-07-18

**Authors:** Anja Rieckert, Ulrike S. Trampisch, Renate Klaaßen-Mielke, Eva Drewelow, Aneez Esmail, Tim Johansson, Sophie Keller, Ilkka Kunnamo, Christin Löffler, Joonas Mäkinen, Giuliano Piccoliori, Anna Vögele, Andreas Sönnichsen

**Affiliations:** 1 Institute of General Practice and Family Medicine, Faculty of Health, Witten/Herdecke University, Alfred-Herrhausen-Str. 50, 58448 Witten, Germany; 2 Department of Medical Informatics, Biometry and Epidemiology, Ruhr University, Universitätsstr. 105, 44789 Bochum, Germany; 3 Institute of General Practice, Rostock University Medical Center, Doberaner Str. 142, 18057 Rostock, Germany; 4 NIHR School of Primary Care Research, University of Manchester, Oxford Road 176, M13 9PL, Manchester, UK; 5 Centre for Primary Care, NIHR School of Primary Care Research, University of Manchester, Oxford Road M13 9PL, Manchester, UK; 6 Duodecim Medical Publications Ltd., Kaivokatu 10 A, 00100 Helsinki, Finland; 7 South Tyrolean Academy of General Practice, Wangergasse 18, 39100 Bolzano, Italy

**Keywords:** Aged, Risk factors, Protective factors, Polypharmacy, Europe, Cross-sectional study, PRIMA-eDS

## Abstract

**Background:**

Polypharmacy is common in older people and associated with potential harms. The aim of this study was to analyse the characteristics of an older multimorbid population with polypharmacy and to identify factors contributing to excessive polypharmacy in these patients.

**Methods:**

This cross-sectional analysis is based on the PRIMA-eDS trial, a large randomised controlled multicentre study of polypharmacy in primary care. Patients’ baseline data were used for analysis. A number of socioeconomic and medical data as well as SF-12-scores were entered into a generalized linear mixed model to identify variables associated with excessive polypharmacy (taking ≥10 substances daily).

**Results:**

Three thousand nine hundred four participants were recruited. Risk factors significantly associated with excessive polypharmacy were frailty (OR 1.45; 95% CI 1.22–1.71), > 8 diagnoses (OR 2.64; 95% CI 2.24–3.11), BMI ≥30 (OR 1.18; 95% CI 1.02–1.38), a lower SF-12 physical health composite score (OR 1.47; 95% CI 1.26–1.72), and a lower SF-12 mental health composite score (OR 1.33; 95% CI 1.17–1.59) than the median of the study population (≤36.6 and ≤ 48.7, respectively). Age ≥ 85 years (OR 0.83; 95% CI 0.70–0.99) led to a significantly lower risk for excessive polypharmacy. No association with excessive polypharmacy could be found for female sex, low educational level, and smoking. Regarding the study centres, being recruited in the UK led to a significantly higher risk for excessive polypharmacy compared to being recruited in Germany 1/Rostock (OR 1.71; 95% CI 1.27–2.30). Being recruited in Germany 2/Witten led to a slightly significant lower risk for excessive polypharmacy compared to Germany 1/Rostock (OR 0.74; 95% CI 0.56–0.97).

**Conclusions:**

Frailty, multimorbidity, obesity, and decreased physical as well as mental health status are risk factors for excessive polypharmacy. Sex, educational level, and smoking apparently do not seem to be related to excessive polypharmacy. Physicians should especially pay attention to their frail, obese patients who have multiple diagnoses and a decreased health-related quality of life, to check carefully whether all the drugs prescribed are evidence-based, safe, and do not interact in an unfavourable way.

**Trial registration:**

This trial has been registered with Current Controlled Trials Ltd. on 31 July 2014 (ISRCTN10137559).

**Electronic supplementary material:**

The online version of this article (10.1186/s12875-018-0795-5) contains supplementary material, which is available to authorized users.

## Background

Older adults (≥65 years) make up an increasing proportion of the European population, particularly the oldest old (≥80 years) [1], and drug use in this age group is common. Depending on setting and age, older adults are prescribed an average of 5.3–6.9 drugs [2–5]. Around 44.2–57.7% of older adults are on ≥5 different drugs, and additional 9.1–23.2% on ≥10 different drugs [4, 6–8]. Concurrent use of multiple medications is known as polypharmacy [9]. However, there is no consensus on the definition of polypharmacy in the literature. Likewise, there is no agreed definition of excessive polypharmacy, though the cut-off point of ≥10 drugs is often used [10].

In some cases, polypharmacy may be inevitable, however, in many patients it appears to be inappropriate [11]. This may be specifically true for older people, as age-related changes in pharmacokinetics and pharmacodynamics increase the risk of adverse drug events [12]. There is some evidence, mostly from observational studies, that polypharmacy in older adults is associated with a number of negative health outcomes such as decreased functional and cognitive health status, increased risk of falls, adverse drug events, hospitalisations, and mortality. However, not all studies found these associations [13]. Risks of adverse drug outcomes increase with an increasing number of medications [14]. Wimmer et al. assessed the association between medication regimen complexity in older people and clinical outcomes, and they concluded that regimen complexity is associated with medication nonadherence and increased rates of hospitalisation [15]. In a retrospective cohort study of adults aged ≥20 years, Payne et al. found an association between unplanned hospital admission and consumption of multiple medications. However, after controlling for multimorbidity only consumption of ≥10 medications was significantly associated with unplanned admissions [16]. Also, the King’s Fund has proposed a cut-off of ≥10 medications as a pragmatic approach to identify polypharmacy patients ‘at risk’, whilst recognising that there is no universal consensus around this [17].

In previous research the association between no-polypharmacy and polypharmacy as well as between no-polypharmacy and excessive polypharmacy was investigated [4, 5, 18–25]. To our knowledge no study so far has analysed possible predictors for excessive polypharmacy in patients consuming multiple drugs. Given the increased risk of adverse health outcomes in older adults taking ≥10 medications it is important to investigate which factors contribute to excessive polypharmacy. We therefore are analysing in this study the independent descriptive variables of the PRIMA-eDS-trial (**P**olypharmacy in chronic diseases: **R**eduction of **I**nappropriate **M**edication and **A**dverse drug events in older populations by **e**lectronic **D**ecision **S**upport) population to identify possible predictors of excessive polypharmacy (using the cut-off of ≥10 substances as proposed above).

## Methods

### Study design and population

This cross-sectional study is based on the baseline data of the PRIMA-eDS trial. In 2015, GPs in five study centres (UK/Manchester, Italy/Bolzano, Austria/Salzburg, Germany 1/Rostock, Germany 2/Witten) were enrolled to recruit patients with polypharmacy. Patient eligibility criteria included age ≥ 75 and taking ≥8 medications regularly. Patient data were collected between September 2014 and September 2015. A more detailed description of the PRIMA-eDS trial has been published [26]. These baseline patient data were used in the analysis presented here.

### Data collection

At each practice the GP or an authorised staff member collected patient data and entered it into an electronic case report form (eCRF; Additional file 1). In the UK, data was also collected at some practices by a regional Clinical Research Network research nurse working with the practice. Baseline data used for this study included: age, sex, height, weight, all drugs with an Anatomical Therapeutic Chemical (ATC) code (prescribed and over the counter), all diagnoses, smoking status, and frailty according to the clinical frailty scale [27]. A health-related quality of life questionnaire (SF-12v2) [28] was also administered to each patient and entered into the eCRF by staff of the study centres. Educational level was recorded according to ISCED-97 which was filled out by the patient [29].

### Outcome and independent variables

According to the King’s Fund [17] and the median number of drugs used by all patients participating in the study, we defined the outcome variables as non-excessive polypharmacy (< 10 substances) and excessive polypharmacy (≥10 substances). Drugs were coded using the ATC [30] classification system of the World Health Organisation and updated to correspond with changes in 2016 and 2017. Each substance was recorded separately. We took into consideration all substances at baseline with an ATC code. Diagnoses were recorded using the ICD-10 code [31].

Independent variables used in the analysis were age, sex, educational level, physical and mental health composite scores derived from the SF-12, frailty status, body mass index (BMI), smoking status, study centre, and number of diagnoses.

### Statistical analysis

Descriptive statistics were used to describe patient demographics and other variables. Means and standard deviations (SD) were used for continuous variables, and proportions for categorical variables. A multivariable generalized linear mixed model was applied to identify factors associated with excessive polypharmacy, with GP practice as a random effect. For the purposes of this analysis, some independent variables were re-categorised: age (into ≥85 years versus < 85); educational level (low versus medium/high); frailty (frail/terminally ill versus managing well/vulnerable); body mass index (BMI ≥30 kg/m^2^ versus BMI < 30 kg/m^2^); smoking status (smoker versus non-smoker/ex-smoker). The number of diagnoses was grouped as >median versus ≤median (> 8 vs ≤8 diagnoses). Physical and mental health composite scores were all grouped as ≤median versus >median, which was for the physical health composite score ≤ 36.635 versus > 36.636 and for the mental health composite score ≤ 48.7 versus > 48.8. Lower physical and mental health composite scores, meaning that they are below the median, imply poorer health-related quality of life. As some variables have a high share of missing data, we performed multiple imputations by fully conditional specification. Cramer’s V was used to look for bivariate associations. A significance level of α = 0.05 was used throughout. Data was analysed using the SAS v9.4 statistical software.

## Results

Three hundred fifty nine GPs and 3904 patients were recruited. Baseline characteristics of the study sample are presented in Tables 1, 2 and 3, in Additional files 2 and 3. A few patients did not meet the inclusion criteria for the randomised controlled trial (see Table 1), but were included for this analysis. On average, participants were taking 10.5 substances (±2.4) and had 9.5 diagnoses (±4.9). HMG CoA reductase inhibitors were the most commonly used drug, followed by proton pump inhibitors, selective beta blocking agents, platelet aggregation inhibitors, ACE inhibitors, sulphonamides, and dihydropyridine derivatives. Among the ten most commonly used substances according to ATC 5 level, pantoprazole was considered as inappropriate according to the EU(7)-PIM list, and for acetylsalicylic acid, bisoprolol, and amlodipine the EU(7)-PIM list recommended an alternative [32]. Essential (primary) hypertension was the most common diagnosis, followed by disorders of lipoprotein metabolism, type 2 diabetes mellitus, and arthrosis.

**Table 1:** Demographic and clinical characteristics of the population

Characteristics	All subjects (*n* = 3904)		Polypharmacy 7–10 substances^b^ (*n* = 1644)		Excessive Polypharmacy ≥10 substances (*n* = 2260)	
	n		n		n	
Sociodemographic data						
Age	3904					
75-85^c^ (n, %)	3036	77.8	1270	77.3	1766	78.1
≥ 85 (n, %)	868	22.2	374	22.7	494	21.9
mean ± SD (years)	3904	81.5 ± 4.4	1644	81.6 ± 4.4	2260	81.5 ± 4.4
Sex	3904					
Female, n (%)	2240	57.4	913	55.5	1327	58.7
Male, n (%)	1664	42.6	731	44.5	933	41.3
Educational level, n (%)	3578^a^					
Low	1536	39.3	669	40.7	867	38.4
Medium	1465	37.5	583	35.5	882	39.0
High	577	14.8	239	14.5	338	15.0
Health-related factors	3736^a^					
Smokers, n (%)	154	3.9	72	4.4	82	3.6
BMI, n (%)	3904					
BMI < 18.5	34	0.9	15	0.9	19	0.8
BMI 18.5–24	957	24.5	435	26.5	522	23.1
BMI 25–29	1606	41.1	710	43.2	896	39.7
BMI ≥30	1307	33.5	484	29.4	823	36.4
Frailty level, n (%)	3781^a^					
Managing well	1643	42.1	804	48.9	839	37.1
Vulnerable	868	22.2	358	21.8	510	22.6
Mildly frail	660	16.9	237	14.4	423	18.7
Moderately frail	505	13.0	153	9.3	352	15.6
Severely frail	97	2.5	32	2.0	65	2.9
Very severely frail	8	0.2	2	0.1	6	0.2
Physical health composite score, median (range)	3484^a^	36.6 (10–68)	1454	39.4 (12–68)	2030	34.7 (10–63)
Mental health composite score, median (range)	3483^a^	48.7 (12–76)	1454	50.3 (14–76)	2029	47.4 (12–74)
Study centre, n (%)	3904					
Austria	587	15.0	259	15.8	328	14.5
Germany 1	981	25.1	351	21.3	630	27.9
Germany 2	742	19.0	334	20.3	408	18.1
Italy	901	23.4	439	26.7	462	20.4
UK	693	17.8	261	15.9	432	19.1
Substances, n (mean ± SD)	3904	10.5 ± 2.4	1644	8.5 ± 0.6	2260	12.0 ± 2.2
Diagnoses, n (mean ± SD)	3898^a^	9.5 ± 4.9	1644	8.2 ± 4.2	2260	10.5 ± 5.2

^a^As n differs from 3904, the rest of the patients have missing data regarding the variable

^b^7 patients took < 7 substances

^c^5 patients were < 75 years old

Legend: *BMI* body mass index, *Germany* 1 Rostock, *Germany* 2 = Witten, *SD* Standard deviation

**Table 2:** Percentage of the population using substances (ATC level 4) according to polypharmacy status

Substances		All subjects		Polypharmacy < 10 substances		Excessive polypharmacy ≥10 substances	
		n	(%)	n	(%)	n	(%)
C10AA	HMG CoA reductase inhibitors	2479	63.5	999	60.8	1480	65.5
A02BC	Proton pump inhibitors	2328	59.6	837	50.9	1491	66.0
C07AB	Beta blocking agents, selective	2240	57.4	909	55.3	1331	58.9
B01AC	Platelet aggregation inhibitors excl. Heparin	1952	50.0	762	46.4	1190	52.7
C09AA	ACE Inhibitors, plain	1751	44.9	792	48.2	959	42.4
C03CA	Sulfonamides, plain	1715	43.9	569	34.6	1146	50.7
C08CA	Dihydropyridine derivates	1611	41.3	658	40.0	953	42.2
C09CA	Angiotensin II antagonists, plain	1355	34.7	505	30.7	850	37.6
C03AA	Thiazides, plain	1354	34.7	555	33.8	799	35.4
A11CC	Vitamin D and analogues	1120	28.7	345	21.0	775	34.3

**Table 3:** Most common diagnoses of the population according to polypharmacy status

Diagnoses		All subjects		Polypharmacy < 10 substances		Excessive polypharmacy ≥10 substances	
		n	(%)	n	(%)	n	(%)
I10	Essential (primary) hypertension	3428	87.8	1426	86.7	2002	88.6
E78	Disorders of lipoprotein metabolism and other lipidaemias	2078	53.2	814	49.5	1264	55.9
E11	Type 2 diabetes mellitus	1850	47.4	686	41.7	1164	51.5
M19	Osteoarthritis	1752	44.9	683	41.6	1069	47.3
I25	Chronic ischaemic heart disease	1473	37.7	566	34.4	907	40.1
M54	Dorsalgia	1442	36.9	499	30.4	943	41.7
I48	Atrial fibrillation and flutter	1172	30.0	471	28.7	701	31.0
I50	Heart failure	1142	29.3	412	25.1	730	32.3
K21	Gastro-oesophageal reflux disease	982	25.2	369	22.5	613	27.1
F32	Depressive episode	853	21.9	292	17.8	561	24.8

### Factors associated with excessive polypharmacy

Table 4 reports the results of the multivariable analysis. Factors were used as described in the methods section. Regarding study centres, Germany 1 as the one having the highest percentage of patients taking ≥10 drugs was chosen as reference for all other centres. Factors significantly associated with excessive polypharmacy were being frail/terminally ill (OR 1.45; 95% CI 1.22–1.71), having more than 8 diagnoses (OR 2.64; 95% CI 2.24–3.11), being obese (OR 1.18; 95% CI 1.02–1.38), having a lower physical health composite score up to the median (OR 1.47; 95% CI 1.26–1.72), and a lower mental health composite score up to the median (OR 1.33; 95% CI 1.17–1.59). In contrast, being ≥85 years old (OR 0.83; 95% CI 0.70–0.99) was significantly associated with a lower risk for excessive polypharmacy. No association with excessive polypharmacy could be found for being female (OR 1.03; 95% CI 0.89–1.19), having a low educational level (OR 0.95; 95% CI 0.80–1.13), and being a smoker (OR 0.85; 95% CI 0.60–1.21). Regarding the study centres, being recruited in the UK led to a significantly higher risk for excessive polypharmacy compared to being recruited in Germany 1 (OR 1.71; 95% CI 1.27–2.30). Being recruited in Germany 2 led to a slightly significant lower risk for excessive polypharmacy compared to Germany 1 (OR 0.74; 95% CI 0.56–0.97). There were no significant associations between recruitment in Austria and Germany 1 (OR 0.97; 95% CI 0.72–1.31) or Italy and Germany 1 (OR 0.90; 95% CI 0.67–1.21).

**Table 4:** Factors associated with excessive polypharmacy (≥10 substances); results from the multivariable generalized linear mixed model

	Univariable		Multivariable	
Factors	OR (95% CI)	*p*	OR (95% CI)	*p*
Sex (female vs male)	1.18 (1.03–1.35)	0.0189	1.03 (0.89–1.19)	0.6718
Age group (≥85 vs < 85)	0.96 (0.82–1.13)	0.6388	0.83 (0.70–0.99)	0.0328
Educational level (low vs medium/high/missing)	0.94 (0.81–1.10)	0.4440	0.95 (0.80–1.13)	0.5506
Frailty (frail/terminally ill vs managing well/vulnerable/missing)	1.83 (1.57–2.13)	<.0001	1.45 (1.22–1.71)	<.0001
BMI (≥30 vs < 30)	1.34 (1.16–1.54)	<.0001	1.18 (1.02–1.38)	0.0303
Smoker (smoker vs non-smoker/ex-smoker/missing)	0.83 (0.59–1.16)	0.2763	0.85 (0.60–1.21)	0.3579
Research centre (Austria vs Germany 1)	0.69 (0.52–0.93)	0.0139	0.97 (0.72–1.31)	0.8519
Research centre (Italy vs Germany 1)	0.58 (0.44–0.75)	<.0001	0.90 (0.67–1.21)	0.4950
Research centre (UK vs Germany 1)	0.92 (0.70–1.22)	0.5561	1.71 (1.27–2.30)	0.0004
Research centre (Germany 2 vs Germany1)	0.71 (0.54–0.94)	0.0150	0.74 (0.56–0.97)	0.0303
Number of diagnoses (> 8 vs ≤8)	2.70 (2.32–3.14)	<.0001	2.64 (2.24–3.11)	<.0001
Physical health composite score (≤36.635 vs > 36.636)	1.83 (1.58–2.11)	<.0001	1.47 (1.26–1.72)	<.0001
Mental health composite score (≤48.7 vs > 48.8)	1.53 (1.33–1.76)	<.0001	1.36 (1.17–1.59)	<.0001

Legend: Germany 1 = Rostock, Germany 2 = Witten

## Discussion

This cross-sectional study is based on a large sample of older patients with polypharmacy recruited for the European randomised controlled multicentre trial PRIMA-eDS. Results suggest that frailty, multimorbidity, and obesity as well as lower physical and mental health composite scores on the SF-12 are independent risk factors for excessive polypharmacy. Also, in multivariable analysis, the country or even the region plays an important role. Old age alone (≥85 years) does not seem to increase the risk of polypharmacy and may even be associated with lower risk. In our study sample, sex, educational level, and smoking status apparently do not contribute to excessive polypharmacy.

### Interpretation and comparison with existing literature

It is easily understandable that > 8 diagnoses contribute to excessive polypharmacy because guideline-adherent treatment of multiple diseases will inevitably lead to a large number of drugs being prescribed. This has also been shown by other studies with no polypharmacy as a comparison to excessive polypharmacy [20, 21, 24].

In our study, being frail/terminally ill is significantly associated with excessive polypharmacy. Saum et al. [33] and Herr et al. [34] showed that taking ≥10 drugs compared to taking ≤4 drugs is significantly associated with frailty [24]. Morley et al. [35] described frailty as a risk factor for a medication increase as physicians lack a concept on how to treat frail old people and thus often start medication. However, the question of causality remains unsettled as polypharmacy may also lead to frailty [36], and polypharmacy as well as frailty may be a result of multimorbidity. Studies suggested that there is a dose-response relationship between the number of drugs taken and the risk of being frail [33, 37].

Regarding health-related quality of life, a lower physical and mental health composite score indicating worse functioning within these health domains are significantly associated with excessive polypharmacy. Jyrkka et al. found moderate and poor self-reported health to be risk factors for excessive polypharmacy compared to no polypharmacy [20]. Polypharmacy patients usually suffer from several diseases and we expect them to have a reduced health-related quality of life due to illness. Here again, causality may not be easily determined as polypharmacy could also lead to a decrease in health-related quality of life e.g. due to adverse effects of drugs.

It is not surprising that obesity is associated with excessive polypharmacy. Obesity has been shown to lead to an increased use of drugs [38] and can result in chronic diseases especially with advancing age [39]. However, a causal inference cannot be made as chronic diseases caused by obesity may be associated with excessive polypharmacy and it might be that obesity is an intermediate variable or a confounder.

We found that being ≥85 years of age is a protective factor against excessive polypharmacy. This was also found by Kim et al. [21] when comparing older patients with and without polypharmacy, and by Onder et al. [24] who detected an inverse correlation between polypharmacy and increasing age. However, Jyrkka et al. [20] found ≥85 years to be a risk factor for excessive polypharmacy compared to no polypharmacy, and two further studies [19, 25] showed being ≥80 years of age to be a risk factor. One explanation for the decreased use of drugs might be that due to a limited life expectancy of these very old people preventive medications are stopped in order to improve the patients’ current well-being [40]. However, whether this really happens is questionable. Age does not influence patients’ priorities in taking preventive medication and reducing adverse events [41], and GPs find deprescribing of preventive medication difficult [42]. Another interpretation could be that excessive polypharmacy patients die earlier and do not reach the very old age.

We did not find that sex was significantly associated with excessive polypharmacy. The literature is conflicting here [19, 20, 24, 25]. In this study there was no significant association between educational level and excessive polypharmacy. In the literature it has been shown that educational level had an impact on health, however, this effect appeared to decrease with age and was not significant anymore in adults ≥51 years [43].

Smoking contributes to the burden of disease. Surprisingly, smoking was not associated with excessive polypharmacy in older polypharmacy patients, but there were very few smokers among the patients in our study.

There was a slightly significant association between excessive polypharmacy and the study centre Germany 1 when compared to Germany 2. One possible explanation could be the differences between the two settings. Germany 1 recruited patients in a more rural setting in the former Eastern part of Germany while Germany 2 recruited patients in the large metropolitan area of the highly industrialised Ruhr-region. These differences cannot be explained by the variables recorded in this study and deserve further investigation. The study centre in the UK was significantly associated with excessive polypharmacy compared to the study centre Germany 1 in the multivariable analysis. A sensitivity analysis showed that the UK was significantly associated with excessive polypharmacy compared to all other centres. Interestingly, the univariable analysis showed a slightly divergent result which was not significant (OR 0.92; 95% CI 0.70–1.22).

The patients in Germany 1 seemed to be frailer and had more diagnoses compared to the patients in the UK. In multivariable analysis the UK resulted in having more excessive polypharmacy. A reversal of effect can result due to the adjustment in the multivariable model. A possible explanation could be the “Quality and Outcomes Framework” (QOF) introduced in 2004 in the UK, which set financial incentives for certain performance indicators (pay-for-performance). Among these were indicators that relate to chronic conditions [44], some of them directly naming the prescription of certain drugs while other indicators indirectly entailed drug treatment in order to reach the targets [45]. Studies observed rising prescription rates of drugs indicated by QOF around the time when the framework was implemented, such as lipid-regulating drugs, renin-angiotensin system drugs [45], ß-blockers or antiplatelet therapy [46].

### Implications

Understanding the health characteristics of an aged population taking several drugs, and investigating factors influencing excessive polypharmacy is highly relevant in times when the geriatric population is growing. This study helps to develop targeted strategies to reduce polypharmacy by identifying factors contributing to excessive polypharmacy. Physicians should especially pay attention to their frail, obese patients that have > 8 diagnoses, check whether all medications are necessary, evidence based and appropriate, and whether there are relevant interactions. To do so, GPs should perform medication reviews for their patients with excessive polypharmacy on a regular basis to optimise these patients’ medication. They should allocate extra time to care for these complex patients which needs to be reimbursed by the health care system.

### Strengths and limitations

The major strength of our study is that we examined a very large sample of older patients representing several different health care settings/countries. We collected various parameters in this geriatric study population which gave us a comprehensive overview of demographic, clinical and functional status, and recorded the frailty level to distinguish between the fitter and the less fit ones. A major limitation of our study is that its cross-sectional design does not allow conclusions on causality. Further limitations are that the health-related quality of life was self-reported and all variables in the eCRF were reported by the GP, by practice staff or by a clinical research nurse. Even though instructions to record patient data were the same throughout all settings, we do not know whether the documentation of variables differs in different settings. True drug consumption is difficult to assess. We instructed the GPs to talk to their patients about all drugs they are taking. However, we were not able to verify drug consumption. Also, in this cross-sectional analysis, only patients were analysed who were recruited according to the inclusion criteria of the PRIMA-eDS trial. We therefore could only investigate patient characteristics associated with excessive polypharmacy in comparison to less excessive polypharmacy as patients without polypharmacy were not included in the trial. Furthermore, external characteristics e.g. of the prescribers could not be taken into account, and we did not judge whether medication intake was appropriate or not.

A further limitation of this study is that multiple relationships between variables exist. Multicollinearity is the cause of conspicuous differences between univariable and multivariable analysis. The interpretation for the affected variables should be regarded with caution. Noticeable correlations were found for the relationship between the research centre and the educational level (Cramer’s V = 0.48), or the number of diagnoses (Cramer’s V = 0.40) respectively, as well as between frailty, the two SF-12 scales (physical health composite score Cramer’s V = 0.20 and mental health composite score Cramer’s V = 0.33) and age (Cramer’s V = 0.22).

The physical and the mental health composite scores as well as frailty were identified as risk factors in both univariable and multivariable analysis, but the ORs are smaller in multivariable analysis because of the dependencies. Frailty and health-related quality of life are closely associated [47], still we retained these variables in our analysis as they measure different concepts.

In univariable analysis, age was not significantly related to polypharmacy. It could be that there is a connection of the variable age with information about the condition of the patient, such as frailty, health-related quality of life, and diagnoses. Frailty [48], lower health-related quality of life [49], and a high number of diagnoses [50] are more common in old age. On the other hand, these factors increase the likelihood of excessive polypharmacy regardless of age [33, 50, 51]. Therefore, the positive effect of high age may become more apparent when adjusting for these factors.

Problematic is the variable “research centre”, which significantly increases the associations found after adjustment for UK/Manchester. This is mainly attributable to the consideration of the number of diagnoses. After adjustment for this variable, the OR increases from 1.10 to 1.71. We did not want to give up the number of diagnoses as an independent variable, as in the literature this is reported as an important risk factor. The variable “research centre” also seemed essential for the model, since this variable represents a variety of influences, such as the quality of the data collection, country-specific features and so on. Yet, it must be regarded with caution.

## Conclusion

Our data suggest that frailty, multimorbidity, obesity as well as low physical and mental health status may be risk factors for excessive polypharmacy. Very old age appears to be a protective factor. Sex, educational level, and smoking are not associated with excessive polypharmacy. To avoid excessive polypharmacy with its possibly unfavourable effects, physicians should carefully review the appropriateness of medication, especially in multimorbid, obese and frail patients.

## Additional files

Additional file 1: Electronic Case Report Form (eCRF) (PDF 201 kb)

Additional file 2: Demographic and clinical characteristics per study centre (PDF 109 kb)

Additional file 3: Percentage of the population using substances (ATC level 5) according to polypharmacy status (PDF 41 kb)

## Abbreviations

ATC: Anatomical Therapeutic Chemical
BMI: Body mass index
CI: Confidence interval
GP: General practitioner
OR: Odds ratio
PRIMA-eDS: Polypharmacy in chronic diseases: Reduction of Inappropriate Medication and Adverse drug events in older populations by electronic Decision Support
QOF: Quality and Outcomes Framework
SD: Standard deviation
UK: United Kingdom
