# Supplementary material for: Polypharmacy in older patients with chronic diseases: a cross-sectional analysis of factors associated with excessive polypharmacy
Source: BMC Fam Pract. 2018 Jul 18;19:113. doi: 10.1186/s12875-018-0795-5 (PMC6052592; doi:10.1186/s12875-018-0795-5)
Supplement: Supplementary file 1 — Electronic Case Report Form (eCRF) (PDF 201 kb) [file 12875_2018_795_MOESM1_ESM.pdf]

Additonal file I Electronic Case Report Form (eCRF)

**Needed patient data/further explanation and information**
**<u>PATIENT IDENTITY</u>**<br><br>○ Internal Study ID<br>○ Year of birth<br>○ Gender (male/female)<br>○ Patient has signed informed consent ➔ tick box for "yes" (this is necessary for entering the following data/ to perform medication review)<br><br>after this section the "new patient" will be created and baseline data can be entered
**<u>CURRENT MEDICATION</u>**<br><br>Please provide current continuous and temporary medication including over-the-counter medication, herbal treatment and treatment prescribed by other medical specialists.<br>You can search directly by the drug's ATC code or by the generic name. Drug brand names are not included.<br>For combination drugs all active components must be entered separately as if they were separate drugs.<br>Exact start dates are not needed at baseline. It is sufficient to use the default value 1.1.2013.<br><br>○ Name + ATC code (out of drop-down menu)<br>○ Frequency (daily - weekly -as needed- other)<br>○ Administration route (oral - rectal -parenteral – topical)<br>○ Start date of medication<br>○ End date and reason for discontinuation (ending a temporary treatment - adverse drug event - after reconsideration - patient´s choice or whish – false entry– other)<br>○ total daily dose + unit
**<u>DIAGNOSES</u>**<br><br>the following list shows the most frequent chronic diseases, please select:<br><br>○ Primary hypertension<br>○ Type2 diabetes<br>○ Hypercholesterolemia<br>○ History of myocardial infarction<br>○ Chronic heart failure<br>○ Chronic ischemic heart disease<br>○ Atrial fibrillation<br>○ Stroke or TIA<br>○ Asthma<br>○ COPD<br>○ Depression<br>○ Insomnia<br>○ Gastroesophageal reflux<br>○ Benign prostatic hyperplasia<br>○ Osteoporosis without fracture<br>○ Arthrosis<br>○ Back pain<br>○ Gout<br>○ Hypothyroidism

- Rheumatoid arthritis

Renal insufficiency is automatically calculated from the GFR.

OTHER DIAGNOSES RELEVANT TO THE MEDICATION

Please provide all permanent diagnoses, current temporary diagnoses and previous diagnoses possibly significant for the treatment.
You can search directly by the ICD-10 code or by the name of the disease in the eCRF.
Exact diagnosis start dates are not needed at baseline. It is sufficient to use the default value 1.1.2013.

- Name + ICD 10 Code as drop down menu
- Start date
- End date (remove diagnosis from the list)
- If an end date is set: Reason for removal (False entry – cured/not active)

SYMPTOMS WITHIN ONE MONTH

Please provide all symptoms compromising the patient's quality of life within the time frame of the previous month by ticking the appropriate box.

- Constipation
- Nausea/ vomiting
- Diarrhea
- Dyspepsia/abdominal discomfort
- Dizziness/vertigo
- Fatigue
- Sleeping problems
- Confusion/delirium
- Pain
- Dyspnea
- Cough
- Rash or itching
- Nycturia
- Leg swelling
- Other (please list)

FALLS

Please enter the number of falls and the severity of injury of falls of this patient during the last 3 months - select out of the following:

- None
- Number of falls with moderate/severe injury

Information on falls

- Serious injury: Medically recorded fracture, head or internal injury requiring accident and emergency or inpatient treatment.
- Moderate injury: Wounds, bruises, sprains, cuts requiring a medical/health professional examination such as physical examination, x-ray, suture.
- Minor injury: Minor bruises or abrasions not requiring health professional assistance; reduction in physical function (e.g. due to pain, fear of falling) for at least three days.
- No injury: No physical injury detected.

MEASUREMENTS AND PROCEDURES

Please provide anthropometric measurements, blood pressure, frailty scale, smoking status and creatinine. Please carry out if not available in your records.
All other lab results are only to be provided if they are available in your records. Otherwise please leave blank as we want to avoid additional laboratory analyses.

- Height
- Weight + date
- BP (Blood Pressure) + date (enter a typical/average BP based on several recent measurements. Use latest measurement if several recent measurements are not available)
- Smoking status (non-smoker - smoker - ex-smoker - not known)
- Creatinine + date
- Total Cholesterol
- HDL Cholesterol
- Triglycerides
- LDL Cholesterol
- Fasting Glucose
- HbA1c + date
- INR + date
- B-Hb + date
- Platelet count + date
- ALT + date
- Potassium + date
- Sodium +date
- Proteinuria (Yes/No)

PROCEDURES (Yes/No)

- Drug Eluting Stent + date
- Transcatheter Aortic Valve Replacement + date
- Heart Valve Replacement mechanical + date

FRAILTY

Frailty scale - Read more about frailty scale on the link on the eCRF or on
http://www.ebmeds.org/cmr/Annex_II_Frailty_scale.pdf

Please select:

- Managing Well: Medical problems well controlled, patient not regularly active beyond routine walking.
- Vulnerable: Not dependent on others for daily help. Being "slowed up", and/or being tired during the day.
- Mildly Frail: More evident "slowed up", and/or being tired during the day, need help in some daily activities (i.e. finances, heavy housework).
- Moderately Frail: Need help with all outside activities/keeping house. Have problems with stairs, need help for bathing and minimal assistance with dressing.
- Severely Frail: Completely dependent for personal care, from whatever cause (physical or cognitive). Not at high risk of dying (within ~ 6 months).
- Very Severely Frail/Terminally Ill: Completely dependent, approaching the end of life (life expectancy < 6 months).
