# Supplementary material for: Polypharmacy in older patients with chronic diseases: a cross-sectional analysis of factors associated with excessive polypharmacy
Source: BMC Fam Pract. 2018 Jul 18;19:113. doi: 10.1186/s12875-018-0795-5 (PMC6052592; doi:10.1186/s12875-018-0795-5)
Supplement: Supplementary file 2 — Demographic and clinical characteristics per study centre (PDF 109 kb) [file 12875_2018_795_MOESM2_ESM.pdf]

Additional file II Demographic and clinical characteristics per study centre

Characteristics	Austria		Germany I		Germany II		Italy		UK	
	**n**		**n**		**n**		**n**		**n**	
**Sociodemographic data**										
Age										
<85 (n, %)	429	73.1	771	78.6	568	76.5	712	79.0	556	80.2
≥85 (n, %)	158	26.9	210	21.4	174	23.5	189	21.0	137	19.8
mean ±SD (years)	587	82.2 ±4.6	981	81.5 ±4.3	742	81.6 ±4.5	901	81.3 ±4.4	693	81.1 ±4.4
Female, n (%)	362	61.7	606	61.8	415	55.9	521	57.8	336	48.5
Educational level, n (%)										
Low	230	39.2	152	15.5	245	33.0	598	66.4	311	44.9
Medium	222	37.8	608	62.0	346	46.6	92	10.2	197	28.4
High	59	10.0	217	22.1	132	17.8	21	2.3	148	21.4
**Health-related factors**										
Smokers, n (%)	11	1.9	45	4.6	23	3.1	44	4.9	31	4.5
BMI, n (%)										
BMI <18.5	5	0.8	8	0.8	8	1.0	7	0.8	6	0.9
BMI 18.524	174	29.6	195	19.9	157	21.2	255	28.3	176	25.4
BMI 25-29	224	38.2	384	39.1	316	42.6	385	42.7	297	42.8
BMI ≥30	184	31.4	394	40.2	261	35.2	254	28.2	214	30.9
Frailty level, n (%)										

Managing well	245	41.7	415	42.3	255	34.4	376	41.7	352	50.8
Vulnerable	124	21.1	229	23.3	178	24.0	170	18.9	167	24.1
Mildly frail	91	15.5	158	16.1	125	16.8	171	19.0	115	16.6
Moderately frail	84	14.3	139	14.2	144	19.4	92	10.2	46	6.6
Severely frail	21	3.6	28	2.9	19	2.6	23	2.5	6	0.9
Very severely frail	2	0.3	2	0.2	4	0.5	0	0.0	0	0.00
Physical health composite score, median (range)	509	36.7 (13-62)	948	35.9 (12-63)	713	35.0 (11-62)	675	38.7 (11-68)	639	37.5 (10-60)
Mental health composite score, median (range)	506	47.6 (16-74)	946	48.6 (12-72)	715	47.5 (17-76)	676	46.8 (14-72)	640	53.5 (16-74)
Substances										
<8	259	44.1	351	35.8	334	45.0	439	48.7	261	37.7
≥10	328	55.9	630	64.2	408	55.0	462	51.3	432	62.3
n (mean ±SD)	587	10.4 ±2.3	981	10.9 ±2.7	742	10.4 ±2.4	901	10.0 ±2.0	693	10.9 ±2.7
Diagnoses, n (mean ±SD)	587	8.5±3.5	981	12.9 ±6.2	742	10.4 ±4.5	901	7.6 ±2.7	693	6.8 ±3.5

Legend: BMI= Body Mass Index, Germany 1= Rostock, Germany 2= Witten, SD= Standard deviation
