# Supplementary material for: Polypharmacy in older patients with chronic diseases: a cross-sectional analysis of factors associated with excessive polypharmacy
Source: BMC Fam Pract. 2018 Jul 18;19:113. doi: 10.1186/s12875-018-0795-5 (PMC6052592; doi:10.1186/s12875-018-0795-5)
Supplement: Supplementary file 3 — Percentage of the population using substances (ATC level 5) according to polypharmacy status (PDF 41 kb) [file 12875_2018_795_MOESM3_ESM.pdf]

Additional file III Percentage of the population using substances (ATC level 5) according to polypharmacy status

**Substances**		**All subjects**		**Polypharmacy <10 substances**		**Excessive polypharmacy ≥10 substances**	
		**n**	**(%)**	**n**	**(%)**	**n**	**(%)**
B01AC06	acetylsalicylic acid[3]	1662	42.6	665	40.5	997	44.1
C10AA01	simvastatin	1597	40.9	650	39.5	947	41.9
C03AA03	hydrochlorothiazide	1243	31.8	514	31.3	729	32.3
C07AB07	bisoprolol[2]	1199	30.7	480	29.2	719	31.8
A02BC02	pantoprazole[1]	1125	28.8	390	23.7	735	32.5
C08CA01	amlodipine[2]	1096	28.1	451	27.4	645	28.5
C09AA05	ramipril	1089	27.9	500	30.4	589	26.1
A11CC05	colecalciferol	1048	8	331	20.1	717	31.7
H03AA01	levothyroxine sodium	931	23.8	338	20.6	593	26.2
C03CA04	torasemide	905	23.2	295	17.9	610	27.0

1= potentially inappropriate medication according to EU(7)-PIM list
2= recommended alternative drug according to EU(7)-PIM list
3= recommended alternative drug (<325 mg) according to EU(7)-PIM list
